# Biomarkers Identifying Tendency to Suicide (BITS): detection of biomarkers in adolescents with suicidal ideation or suicidal behavior for early prevention or intervention. A prospective cohort study

**DOI:** 10.3389/fnhum.2026.1721827

**Published:** 2026-01-26

**Authors:** Marika Orlandi, Francesca Marazzi, Chiara Morandi, Matteo Gastaldi, Renato Borgatti, Martina Maria Mensi

**Affiliations:** 1 Department of Brain and Behavioral Sciences, University of Pavia, Pavia, Italy; 2 Child Neurology and Psychiatry Unit, IRCCS Mondino Foundation, Pavia, Italy; 3 Neuroimmunology Laboratory and Neuroimmunology Research Section, IRCCS Mondino Foundation, Pavia, Italy

**Keywords:** assessment, biomarkers, blood–brain barrier, HPA, inflammation, prevention, suicidal attempts, suicidal ideation

## Abstract

Adolescent suicidality represents a major and persistent public health concern, with sustained increases in suicidal ideation and behaviors observed in recent years. Although suicide risk is widely conceptualized as a complex and multifactorial phenomenon, biological vulnerability factors remain insufficiently integrated into adolescent clinical assessment frameworks. In particular, neuroinflammatory processes, hypothalamic–pituitary–adrenal (HPA) axis dysregulation, and alterations in blood-brain barrier integrity have emerged as potentially relevant but underexplored contributors to suicidality. This prospective clinical cohort study adopts an integrative, multi-method approach to enhance the characterization of suicidality among help-seeking adolescents. We hypothesize that adolescents with suicidal ideation or suicidal behaviors will show distinct peripheral biomarker profiles compared with adolescents without suicidal concerns. The primary analyses focus on group differences at baseline in inflammatory cytokines, markers of systemic inflammation, HPA-axis activity, and blood-brain barrier-related biomarkers, as well as measures of suicidality severity and global functioning. Secondary outcomes include associations between biomarker levels and psychopathological severity and functioning, and longitudinal changes in biological and clinical measures, including transitions in suicidality status, over a 2-month follow-up. Participants will undergo a comprehensive psychodiagnostic assessment and standardized peripheral blood sampling at baseline and follow-up. By integrating biological measures with detailed psychological and behavioral profiling within a longitudinal design, this protocol aims to establish a framework for biomarker-informed risk stratification of high-risk adolescents, ultimately informing more precise and developmentally sensitive suicide prevention strategies.

## 1 Introduction

Mental health issues represent a significant public health concern during adolescence. Although the acute phase of the COVID-19 pandemic has ended, recent studies continue to document its lasting impact on youth psychological wellbeing, with persistent increases in psychiatric symptoms, including suicidal ideation and self-harming behaviors (Mensi et al., 2021, 2022; Wong et al., 2021; Vloo et al., 2021; Pizarro-Ruiz and Ordóñez-Camblor, 2021; Newlove-Delgado et al., 2021). According to the World Health Organization (WHO), the act of deliberately taking one's own life remains the leading cause of death worldwide and the third leading cause of death among individuals aged 15–29 years (World Health Organization, 2024). Within the broader term ‘suicidality', it is essential to clarify the distinctions between its core components. In particular, suicidal ideation (i.e., thoughts concerning death or self-harm) and suicidal behaviors (including actual, interrupted, or aborted attempts) follow the standardized operational definitions provided by the Columbia-Suicide Severity Rating Scale (C-SSRS) (Posner et al., 2011). Suicide prevention in adolescents must therefore be prioritized within public health policy agendas (Morcillo et al., 2025).

The increasing number of adolescents exhibiting suicidal ideation or engaging in self-harming behaviors, including attempted, interrupted, or aborted suicides, underscores the need to improve both diagnostic pathways and the clinical management of adolescents and their families, particularly during acute phases of crisis (Coci et al., 2022). Considering suicide as a complex phenomenon resulting from the interplay of multiple biological, psychological, and social factors (Nock et al., 2010; Cha et al., 2018), it is reasonable to assume that risk stratification should consider all these dimensions. At present, however, suicide risk assessments primarily focus on psychological and occasionally social factors, while the potential contribution of biological markers has been relatively underexplored. Integrating biomarker analysis with psychological dimensions may enhance early identification of adolescents at elevated suicide risk-related phenotypes and allow for a more precise characterization of distinct risk profiles. In particular, few studies have combined a broad panel of peripheral biomarkers with a comprehensive, multi-method psychological assessment within a longitudinal design in a clinically well-characterized adolescent sample. This integrative approach enables investigation of how biological vulnerability markers interact over time with psychological functioning, symptom severity, and clinical trajectories, thereby addressing a critical gap between human neuroscience research and real-world clinical practice.

Among the most promising approaches is the investigation of peripheral biomarkers. Some authors have demonstrated a correlation between chronic inflammation, both central and peripheral, and a range of neuropsychiatric disorders (Bauer and Teixeira, 2019), as well as the presence of a distinct “inflammatory signature” in individuals who died by suicide, regardless of their specific psychiatric diagnoses. Several recent studies have explored the role of inflammation and the immunological underpinnings of suicidality (Serafini et al., 2020), with a particular focus on peripheral cytokines. For instance, pro-inflammatory biomarkers such as interleukin (IL)-6, IL-8, white blood cell count, polymorphonuclear leukocyte count, and the neutrophil-to-lymphocyte ratio (NLR) have been associated with increased suicide risk when compared to individuals diagnosed with depression without suicidal ideation (Keaton et al., 2019; Knowles et al., 2019; Daray et al., 2024).

Other authors focusing on pediatric populations have reported significantly increased mRNA and protein expression levels of IL-1β, IL-6, and tumor necrosis factor-alpha (TNF-α) in individuals who died by suicide compared to controls (Pandey et al., 2012). Additionally, elevated IL-6 levels have been observed in children who experienced clinical deterioration following fluoxetine treatment (Amitai et al., 2020). Two previous meta-analyses further confirmed that circulating levels of IL-1β and IL-6 are strongly associated with suicidality and may serve to distinguish suicidal from non-suicidal patients (Black and Miller, 2015; Ducasse et al., 2015). Elevated levels of C-reactive protein (CRP) have also been consistently linked to suicidal ideation, suicide attempts, and death by suicide (Miola et al., 2021; Chen et al., 2020).

Moreover, other biomarkers have shown promise as potential indicators of suicide risk, especially in cases where inflammation crosses the blood–brain barrier and causes neurotoxicity. These include S100B, intercellular adhesion molecule-1 (ICAM-1), vascular cell adhesion molecule-1 (VCAM-1), platelet-derived growth factor receptor (PDGF-R), and neurofilaments (NFL) (Falcone et al., 2010; Ramezani et al., 2022; Yen et al., 2022). Notably, cerebrospinal fluid levels of IL-6 are significantly higher in individuals at risk of suicide compared to controls (Lindqvist et al., 2009). Lastly, dysregulation of the hypothalamic–pituitary–adrenal (HPA) axis, particularly when combined with psychosocial stress and psychological distress, has emerged as a potential contributing factor to suicidality (Morena et al., 2024).

However, findings on biological correlates of suicidality in adolescents are highly heterogeneous, often influenced by comorbid psychiatric conditions, psychopharmacological treatments, and methodological differences across studies, including sample size, study design, and definitions of suicidal ideation (SI) vs. suicidal behavior (SB). Many studies did not adequately control for these confounders, and the overlap between SI and SB participants adds further complexity, potentially biasing results. Associations between peripheral immune markers and suicidality have been reported, but causality remains unclear, and their clinical significance is still to be established (Daray et al., 2024; Pak et al., 2025). Moreover, while evidence points to a peripheral inflammatory state in at-risk youth, it is uncertain to what extent these peripheral changes reflect central nervous system alterations. Recent meta-analytic evidence in both youth and adult populations suggests that leukocyte and neutrophil increases can occur in both peripheral blood and cerebrospinal fluid, supporting a possible systemic-central link (Gigase et al., 2023). Yet the pathway connecting peripheral inflammation, blood–brain barrier disruption, and CNS effects on suicidal ideation and behavior remains underdeveloped. Overall, these limitations highlight the need for longitudinal, multi-method studies that carefully account for confounders to clarify the role of biomarkers in adolescent suicidality.

The present study aims to expand the existing scientific literature by documenting cases of suicidal ideation and suicidal behaviors in adolescents. This research seeks to characterize this population not only from a psychological and behavioral perspective but also through a neuroinflammatory lens, providing a more comprehensive understanding of suicide risk. Emerging evidence suggests that elevated levels of peripheral inflammatory biomarkers (e.g., IL-6, TNF-α, CRP) are associated with suicidality in multiple clinical populations, supporting the hypothesis that systemic inflammation may be linked to suicide risk (Baldini et al., 2025). By simultaneously assessing inflammatory markers, HPA-axis activity, and indicators of blood–brain barrier integrity, this study is grounded in contemporary neurobiological models of suicidality that conceptualize suicide risk as the result of stress-related immune activation, neuroendocrine dysregulation, and altered brain–periphery signaling. Integrating these biological dimensions with detailed psychological and behavioral profiling may help refine and empirically test these models in a developmental clinical population.

Although biomarker analysis requires initial investment, a recent systematic review highlighted that suicide attempts impose substantial healthcare costs, ranging from $36 to $644 per person per emergency admission, up to $621,166 per death (Jain et al., 2024). In light of that, the use of biomarkers may support the selection and timing of preventive and therapeutic interventions tailored to adolescents at elevated risk, ultimately decreasing hospitalization rates, illness duration, and healthcare costs. Finally, given that suicidality is a transdiagnostic and widespread issue across populations, it is crucial to disseminate these findings to raise public awareness and influence more effective prevention strategies.

Within this framework, the study seeks to explore how psychological, behavioral, and neuroinflammatory characteristics converge in adolescents exhibiting suicidal ideation or behaviors, addressing a critical gap in the literature: longitudinal data on the interplay between peripheral inflammatory markers, blood-brain barrier integrity, HPA-axis activity, and clinical evolution in help-seeking adolescents are currently lacking. By examining the interplay between these dimensions, we aim to delineate distinct risk profiles and better understand how integrated biological and psychological measures can enhance the early detection of high-risk individuals. Ultimately, this approach may inform the development of more precise and timely intervention strategies, improving both clinical outcomes and the effectiveness of suicide prevention efforts.

The study addresses three main research questions:

1. Do biomarker levels differ significantly between suicidality groups at T0?
2. Which biomarkers correlate with psychopathological severity and functioning in help-seeking adolescents?
3. Do biomarker profiles and suicidality group membership change from T0 to T1?

## 2 Methods and analysis

### 2.1 Study design

This clinical prospective cohort study will be conducted in accordance with the Strengthening the Reporting of Observational Studies in Epidemiology (STROBE) Statement and following the Declaration of Helsinki (1964) and its later amendments (World Medical Association, 2025). The Lombardy Territorial Ethics Committee 6, Italy, approved this study (2023-3.11/107). The participants' parents or caregivers will provide written informed consent and will be free to withdraw from the study at any time. Patients will provide informed consent to participate in the study and to the processing of their data. Adolescents cannot participate without caregiver consent. Participants and caregivers do not receive financial reimbursement. They will receive standard care independently of their participation in the study.

The dataset generated and analyzed in this study will be de-identified and pseudonymized and made available in the Zenodo repository.

Participation in the study could involve possible risks related to venous blood sampling, therefore, medical and nursing supervision is required during the study.

The study will last 4 years, and participants will be evaluated at admission (T0) and after 2 months (T1). The 2-month follow-up aims to balance feasibility, minimize attrition, and ensure completion of longitudinal assessments. The study will continue for the planned duration and is not limited to a fixed number of participants. This follow-up can help clarify the effect that a multidisciplinary, multimethod assessment and, consequently, referral to the service most appropriate to the patient's needs, has on suicide risk progression over time.

### 2.2 Participants

The study will involve help-seeking adolescents aged 12–18 years (inclusive) admitted as inpatients or day-hospital to the Child Neuropsychiatry Unit of the third-level IRCCS Mondino Foundation in Pavia, Italy. Table 1 summarizes the exclusion criteria, and Table 2 shows the strategy adopted to manage major clinical and biological confounders. Given the severity and clinical complexity of the sample, several factors (e.g., psychotropic medication use and psychiatric comorbidities) were not used as exclusion criteria but were systematically recorded and controlled for.

**Table 1:** Exclusion criteria.

**Domain**	**Exclusion criteria**	**Rationale**
Age	Age < 12 or >18 years	Outside target developmental range
Language/comprehension	Insufficient understanding of the Italian language	Inability to complete interviews and self-report measures reliably
Cognitive functioning	Intellectual disability (IQ ≤ 70)	Limits validity of psychological assessment and self-report
Psychiatric conditions	Current active psychosis or acute manic episode	Severe psychopathology likely to profoundly affect biomarker levels and assessment validity
Neurological conditions	Known neurological disorders (e.g., epilepsy, traumatic brain injury, neurodegenerative diseases)	Potential direct effects on neuroinflammatory and neurodegenerative biomarkers
Medical conditions	Autoimmune diseases, chronic inflammatory conditions	Direct confounding effects on immune and inflammatory markers
Acute medical illness	Acute infectious illness within the previous 4 weeks	Transient immune activation affecting biomarker validity
Medication affecting biomarkers	Systemic corticosteroids or immunosuppressive agents at the time of sampling	Strong direct effects on inflammatory and HPA-axis markers
Consent	Lack of written informed consent from both adolescent and caregiver	Ethical requirement

**Table 2:** Potential confounders.

**Factor**	**Management strategy**
Psychotropic medications (e.g., antidepressants, antipsychotics, mood stabilizers)	Fully recorded (type, dose, duration); included as covariates in statistical analyses
Psychiatric comorbidities	Assessed using K-SADS-PL DSM-5; included as covariates
Sex assigned at birth	Included as covariate
Age at baseline	Included as covariate
Socioeconomic status (SES)	Recorded and explored as potential covariate
Incomplete follow-up	Addressed using linear mixed-effects models

### 2.3 Procedures and measures

#### 2.3.1 Primary aim

The primary aim is to characterize adolescents with suicidal ideation and suicidal behaviors by integrating psychological, behavioral, and neuroinflammatory assessments, and to identify baseline differences in biomarker profiles between three suicidality groups.

At first admission to our Institute (T0), clinicians will collect sociodemographic information, including age at baseline, sex assigned at birth, gender identity, ethnicity, and socioeconomic status (SES) (Hollingshead, 1975), as well as current and past medications (type, dose, and duration) and comorbid psychiatric or medical conditions. Then, intellectual functioning will be assessed, and the presence of intellectual disability (IQ ≤ 70) will be excluded using the Wechsler Intelligence Scale for Children-Fourth Edition (WISC-IV) (Wechsler, 2003), the Wechsler Intelligence Scale for Children-Fifth Edition (WISC-V) (Pezzuti et al., 2024) for adolescents less than 16 years old, or the Wechsler Adult Intelligence Scale-Fourth Edition (WAIS-IV) (Wechsler, 2008) for participants aged 16 years and older. Then, subjects eligible to participate in the study will be divided into three groups based on their responses to the C-SSRS regarding the last 6 months.

C-SSRS is a semi-structured clinical interview that assesses suicidal ideation severity, lifetime, and in the past 6 months. It uses a scale from 1 to 5 with dichotomous answers, yes or no (1 = wish to be dead, 2 = non-specific active suicidal thoughts, 3 = active suicidal ideation with any method, or plan, without intent to act, 4 = active suicidal ideation with some intent to act, without a specific plan, and 5 = active suicidal ideation with detailed plan and purpose). It also explores the intensity of suicidal ideation, suicidal attempts (actual, interrupted, aborted, preparatory acts or behavior, and suicidal behavior during the assessment period), and the number of those attempts. It has excellent internal consistency and reliability (Posner et al., 2011; Mundt et al., 2010; Nam et al., 2024).

The first group will include adolescents who will present suicidal ideation without suicidal behavior or previous suicide attempts (SI), i.e., who will have obtained at least one “yes” response to answers 1 through 5 in the “Suicidal Ideation” section of the C-SSRS scale related to the presence of suicidal ideation, but who will have answered “no” to questions in the “Suicidal Behavior” section of the C-SSRS scale associated with the presence of actual attempts, interrupted attempts and/or aborted attempts.

The second group will consist of adolescents who have exhibited a previous history of suicidal behavior (actual non-fatal, interrupted, or aborted suicide attempt) (SB), i.e., who will have obtained at least one “yes” response in the “Suicidal Behavior” section of the C-SSRS scale regarding the presence of concrete attempts, aborted attempts, and/or failed attempts.

The third group will include those adolescents without any suicidal concern (NSC), i.e., who will not have answered “yes” to any question on the C-SSRS, either in the “Suicidal Ideation” section or in the “Suicidal Behavior” section.

To explore the neurobiological mechanisms potentially underlying suicidality in adolescence, peripheral blood samples will be collected from all participants at T0. Participants will be stratified into the three groups as mentioned before.

The biological analyses will focus on biomarkers frequently associated with neuroinflammation, HPA axis regulation, infectious stress, and the integrity of the blood-brain barrier. Plasma levels of IL-6, IL-1β, and TNF-α will be measured to evaluate immune-inflammatory activation. Functioning of the HPA axis will be assessed by quantifying serum concentrations of adrenocorticotropic hormone (ACTH) and cortisol. Markers of systemic stress and low-grade inflammation, including CRP, monocyte-to-lymphocyte ratio (MLR), and NLR, will be derived from complete blood counts and high-sensitivity CRP assays. In addition, serum or plasma markersof neurovascular integrity and potential blood-brain barrier disruption, such as S100B, ICAM-1, VCAM-1, PDGF-R, and NFL, will be quantified using validated immunoassay techniques, including enzyme-linked immunosorbent assays (ELISA). To clarify the rationale for each biomarker, Table 3 summarizes the hypothesized role, relevant pathophysiological pathway, and the planned assay method for each marker.

**Table 3:** List of biomarkers, their role, and assay methods.

**Biomarker**	**Hypothesized role**	**Assay method**
IL-6	Pro-inflammatory cytokine linked to suicidality	ELISA
IL-1β	Neuroinflammation	ELISA
TNF-α	Systemic inflammation	ELISA
CRP	Low-grade inflammation/stress	High-sensitivity immunoassay
ACTH	HPA axis activation	Immunoassay
Cortisol	HPA axis dysregulation	Immunoassay
S100B	Blood–brain barrier integrity/neurotoxicity	ELISA
ICAM-1	Neurovascular adhesion, BBB disruption	ELISA
VCAM-1	Neurovascular adhesion, BBB disruption	ELISA
PDGF-R	Neurovascular and glial signaling	ELISA
NFL	Axonal damage/neurodegeneration	ELISA
MLR	Systemic immune balance	CBC-derived
NLR	Systemic immune balance	CBC-derived

Venous blood samples will be collected from all participants under fasting conditions and strictly between 7:00 and 9:00 AM, to minimize circadian variability. In cases of non-fasting or off-window blood sample collection, the blood draw will be rescheduled and repeated within a maximum of 1 week. Any non-conforming sample will be discarded and not used for biomarker analyses. Samples will be collected into EDTA tubes and serum-separation tubes, depending on the type of analysis. The samples will be centrifuged promptly after collection and stored at −80 °C within 2 h. To ensure analytical consistency, stability, and comparability of results across groups, all biochemical assays will be performed in batch mode once the number of collected samples is sufficient to saturate assay plates, thereby minimizing inter-assay variability.

All procedures related to sample collection, handling, transportation, and storage adhere to the Standard Operating Procedures (SOPs), and all personnel are trained in accordance with them. Samples showing signs of degradation or contamination (e.g., hemolysis) will be excluded from analyses.

Biomarkers will be analyzed only if sample quality, availability, and assay feasibility permit. Analyses will therefore focus primarily on markers for which complete and reliable data will be available.

The laboratory performing biomarker analyses is fully blinded to participants' clinical information, group allocation, and psychodiagnostic results.

To assess participants' characteristics, a multi-method tiered assessment will be conducted. Trained child and adolescent neuropsychiatry residents and psychologists perform all psychodiagnostic assessments. Interviewers have specific training on each instrument, and supervision is provided to ensure standardized administration.

All participants complete a core set of measures, which are essential for group stratification and clinical characterization:

- To evaluate cognitive functioning, we will use the age-appropriate Wechsler scale (Wechsler, 2003; Pezzuti et al., 2024; Wechsler, 2008), as mentioned before.
- C-SSRS to define suicidal ideation/behavior groups,
- The Schedule for Affective Disorders and Schizophrenia for School-Age Children-Present and Lifetime Version (K-SADS-PL DSM-5) (Kaufman et al., 2019, 2016), a diagnostic interview for the assessment of psychopathological disorders (past and current) in children and adolescents according to DSM-5 criteria. K-SADS is administered separately to patients and parents, and the clinician merges the interviews to obtain the most accurate information. Interviews are conducted in separate sessions for children and caregivers to ensure independent reporting.
- The Children's Global Assessment Scale (CGAS) (Shaffer et al., 1983), a clinician-report scale that assesses the individual's psychosocial and occupational functioning on a 100-point scale ranging from mental health to severe mental disorder with risk of death.
- The Clinical Global Impression (CGI) (Guy, 1976), the most widely used scale for global assessment of psychopathology. It allows global judgment regarding the severity of illness, global improvement, and therapeutic efficacy index.

Additional instruments are included to provide complementary dimensional and personality profiling, which enrich the characterization of suicidality beyond core diagnostic measures.

- The Structured Clinical Interview for DSM-5 Personality Disorders (SCID-5-PD) (First et al., 2017), a semi-structured diagnostic interview that assesses personality disorders according to DSM groups and diagnostic criteria. It can be used to make categorical (present or absent) or dimensional diagnoses of personality disorders. It will be administered to participants aged 14 years and above.
- The Multi-Attitude Suicide Tendency scale (MAST) (Orbach et al., 1991), a self-report questionnaire dedicated to tendencies regarding life and death in developmental age. It is composed of four subscales that explore subjective attitudes as mediators of suicidality (Repulsion by death, Attraction toward life, Repulsion by life, and Attraction toward death.
- The Minnesota Multiphasic Personality Inventory-Adolescent (MMPI-A) (Butcher et al., 1992) is a self-report used for personality assessment in adolescents. It enables clinicians to identify psychiatric, clinical, and neuropsychological disorders in adolescence, as well as the adolescent's emotional adjustment.
- The Rorschach Performance Assessment System (R-PAS) (Meyer et al., 2011), a perceptual test that, through the administration of 10 inkblot tables, allows the examiner to have a comprehensive assessment of the person's mental functioning and personality structure. The task creates an associative problem-solving situation that facilitates the projection of internal content. R-PAS scoring follows standardized administration, coding, and interpretation protocols, including both raw and complexity-adjusted scores. The system evaluates multiple domains, such as perceptual accuracy, thought organization, stress and distress, coping strategies, and interpersonal functioning. R-PAS is the most up-to-date, statistically valid, and reliable method, and reflects international evidence (Meyer et al., 2014; Pignolo et al., 2017; Viglione et al., 2022).
- The Beck Inventory Scale—Second edition (BDI-II) (Beck et al., 2011), a self-assessment tool for adolescents and adults that assesses the severity of symptoms characterizing depressive disorder in accordance with DSM diagnostic criteria.
- The Child Behavior Checklist (CBCL 6-18) (Achenbach and Rescorla, 2001) is a questionnaire inspired by the DSM diagnostic categories, completed by parents or caregivers, that assesses the social skills, emotional, and behavioral problems of children and adolescents aged 6–18 years.
- The Beck Hopelessness Scale (BHS) (Beck and Steer, 1988) is a self-report instrument consisting of 20 statements with dichotomic true/false responses, formulated to measure, in the previous week, the extent of negative or positive views about the future.
- The Youth Self-Report (YSR 11-18) (Achenbach, 1991), a parallel version of the CBCL that is completed in person by 11–18-year-olds.

Inpatient assessments are conducted over several days (7–10 days) to allow completion without overburdening participants. Day-hospital assessments are conducted over 1–2 nonconsecutive days and focus on core measures. Standard clinical care includes cognitive testing (age-appropriate Wechsler scales), the K-SADS, the CGI, the CGAS, the CBCL, and the YSR. Additional evaluations (e.g., SCID-5 PD, MMPI-A) and performance tests (e.g., R-PAS) are administered quite frequently to confirm and support the diagnoses. Some additional questionnaires are administered for research purposes (e.g., MAST, BHS).

#### 2.3.2 Secondary aim

We also aim to investigate longitudinal changes in biomarker levels and psychological/behavioral measures from T0 to T1, examining associations with shifts in suicidality group membership and symptom severity, while exploring correlations between biomarkers and clinical, cognitive, and overall functioning. All participants will be assessed at T0 and at 2 months after discharge. The 2-month follow-up is intended to capture short-term clinical and biological changes following acute assessment and care allocation. At T1, only measures necessary for longitudinal assessment of symptom change and suicidality trends (MAST, BDI-II, BHS, CBCL, YSR, CGI, and CGAS) are reassessed, minimizing participant burden. A blood draw will be taken at T1 for reevaluation of neuroinflammatory processes. T0 biomarker results are not available to assessors at T1. Membership in one of the three groups will be reevaluated at this stage using the C-SSRS “since the last visit” version, and the trend in the marker profile will also be analyzed according to these changes.

### 2.4 Data analysis

#### 2.4.1 Sample size and power

Given the paucity of longitudinal biomarker studies specifically focused on suicidality in adolescents, precise effect-size estimates are not available to inform formal sample size calculation. Therefore, the present study adopts a conservative and exploratory approach to power estimation. We estimated the sample size to detect group differences in multivariate biomarker profiles across three suicidality groups (SI, SB, NSC) at T0. Assuming a small-to-moderate effect size (*f* = 0.25), a power of 80%, and a two-sided significance level of α = 0.05, a total sample size of approximately 180 participants is required for multivariate group comparisons. This estimation is conservative, as the primary analyses will employ MANOVA/MANCOVA and linear mixed-effects models, which increase statistical efficiency by accounting for correlations among biomarkers and repeated measurements within individuals.

To account for anticipated attrition over the follow-up period, estimated at approximately 20–25% in this clinical population, the planned recruitment target was increased to approximately 225 adolescents. The use of linear mixed-effects models will further allow the inclusion of participants with incomplete follow-up data, minimizing the impact of attrition on statistical power.

#### 2.4.2 Planned statistical analysis

Statistical analysis will be performed using R (Posit Team, 2025). Descriptive statistics will be used to summarize clinical, psychological, and biological variables and will be presented as means and standard deviations (or median and quartiles, if appropriate). Categorical variables will be presented as frequencies and percentages. The Shapiro-Wilk test will be performed to check the normality of the data.

An exploratory MANOVA/MANCOVA will be used to examine overall biomarker profiles across groups, adjusting for relevant covariates, such as age and sex at birth.

At T0, group differences among the SI, SB, and NSC groups will be examined. Differences in psychological test scores and biomarker levels between groups will be analyzed using ANOVA or the Kruskal-Wallis test, depending on the distribution of the data. In case of significance, *post-hoc* pairwise comparisons will be conducted and adjusted for multiple comparisons.

Moreover, dimensional analyses will be conducted using continuous suicidality severity scores derived from the C-SSRS, as well as measures of psychopathological severity and functioning. Associations between biomarkers and psychological variables will be examined using correlation analyses and multivariable linear regression models, adjusting for relevant covariates including age, gender at birth, medications, and comorbid diagnoses.

Longitudinal changes in biomarker levels and clinical measures from T0 to T1 will be examined using linear mixed-effects models with random intercepts for participants to account for within-subject correlation. Fixed effects will include time, group, and their interaction, with age, gender at birth, medications, and comorbid diagnoses as covariates. This approach allows for the inclusion of participants with incomplete follow-up data.

Changes in suicidality group membership over time, as defined by C-SSRS scores, will be analyzed using mixed-effects logistic regression models to assess the association between biomarker levels and transitions in suicidality status (e.g., from SI to SB or remission). When group imbalance occurs, effect sizes and confidence intervals will be emphasized rather than relying solely on statistical significance.

Sensitivity analyses will also be conducted using alternative groupings (any suicidality vs. no suicidality) to assess the robustness of findings.

## 3 Anticipated limitations and future directions

This study aims to deepen knowledge about adolescent suicidality by combining psychodiagnostic assessments with the analysis of biological markers of neuroinflammation and stress, thereby characterizing both suicidal ideation and suicidal behaviors in this population.

This study has some anticipated limitations. First, although we aim to investigate biomarkers specifically associated with suicidality, it is possible that observed alterations, even if accounting for covariates, may reflect general psychological distress or other psychiatric conditions rather than suicide-specific processes, considering that suicide ideation and behaviors are transdiagnostic. Second, the study population consists of help-seeking adolescents who choose to participate in the study in an institution specialized in severe psychopathological disorders and may not be representative of the broader adolescent population, potentially limiting generalizability. Third, psychotropic medication use is common in this population and may influence inflammatory and neuroendocrine markers. Although medication type, dose, and duration will be carefully recorded and included as covariates, residual confounding cannot be fully excluded, and causal inferences regarding biological mechanisms are not possible given the observational design. Fourth, gender-related differences in help-seeking behavior, psychopathology, and suicidality may further confound associations between biomarkers and clinical outcomes. Finally, despite efforts to standardize sample collection and psychodiagnostic assessment, limitations such as participant burden, variability in test completion, follow-up duration, and follow-up adherence may introduce missing data or bias. To conclude, the protocol is designed for a tertiary-care setting and is intended as a reference framework for future streamlined approaches.

Future research may extend follow-up durations and further explore biological aspects, such as genetic and epigenetic factors, as well as other psychopathology characteristics. Furthermore, while integrating biological data into routine clinical assessments holds promise for supporting early identification of adolescents at elevated risk for suicidality, it is important to note that translation into clinical practice represents a long-term goal that requires further validation and interventional studies. Moreover, the study's findings may contribute to the development of standardized diagnostic protocols and to the improvement of adolescent mental health services. Finally, this research has the potential to enhance care also for caregivers and families, ensuring that clinical and psychosocial needs are adequately addressed.

## 4 Ethics and dissemination

The present study has been designed and will be conducted in accordance with the Declaration of Helsinki and its subsequent amendments. Ethical approval was obtained from the Lombardy Territorial Ethics Committee 6 (approval number: 2023-3.11/107). Before enrollment, detailed information regarding the study's aims, procedures, potential risks, and benefits will be provided to both the adolescent participants and their legal guardians. Written informed consent will be obtained from parents or legal guardians, and adolescents will provide their informed assent. Participation will be entirely voluntary, and participants can withdraw at any stage without any consequences for their ongoing clinical care.

Given the vulnerability of the target population, which consists of adolescents exhibiting suicidal ideation or behaviors, the study protocol incorporates stringent safeguards to protect participants' mental and physical wellbeing. Continuous medical and psychological monitoring will be ensured throughout the study in order to address any adverse events or distress promptly. In the event of clinical deterioration or emergent risk, appropriate interventions will be immediately facilitated by the multidisciplinary clinical team at the Child Neurology and Psychiatry Unit of the IRCCS Mondino Foundation.

All participant data will be handled in accordance with the General Data Protection Regulation (GDPR) 2016/679 and applicable Italian privacy legislation. Data will be pseudonymized prior to any analysis to preserve participant confidentiality, and access to personal identifiers will be restricted exclusively to authorized research staff. Biological samples will be stored securely and managed following institutional biobank protocols to maintain specimen integrity and comply with biosafety standards.

Risks related to venous blood collection, such as localized pain, bruising, or transient discomfort, will be minimized by qualified clinical personnel operating under established standard operating procedures. Any adverse effects will be diligently documented and reported to the Ethics Committee.

Dissemination of findings will adhere to the principles of open scientific communication and public health impact. Study results will be submitted for publication in peer-reviewed journals and presented at relevant national and international conferences. Moreover, efforts will be made to share insights with clinical practitioners and mental health stakeholders through seminars and workshops aimed at optimizing adolescent suicide prevention strategies and informing service-level planning and future policy discussions. Where appropriate, anonymized datasets will be deposited in open-access repositories such as Zenodo to foster transparency and reproducibility. Participants and their families who wish to receive summaries of the overall study will receive outcomes in accessible, non-technical language.
